# *FepR* as a Central Genetic Target in the Adaptation to Quaternary Ammonium Compounds and Cross-Resistance to Ciprofloxacin in *Listeria monocytogenes*

**DOI:** 10.3389/fmicb.2022.864576

**Published:** 2022-05-18

**Authors:** Pierre-Emmanuel Douarre, Yann Sévellec, Patricia Le Grandois, Christophe Soumet, Arnaud Bridier, Sophie Roussel

**Affiliations:** ^1^Maisons-Alfort Laboratory for Food Safety, Salmonella and Listeria Unit, University of Paris-Est, French Agency for Food, Environmental and Occupational Health and Safety (ANSES), Maisons-Alfort, France; ^2^Antibiotics, Biocides, Residues and Resistance Unit, French Agency for Food, Environmental and Occupational Health and Safety (ANSES), Fougères, France

**Keywords:** *Listeria monocytogenes*, biocide adaptation, ciprofloxacin, cross-resistance, *fepR* gene, benzalkonium chloride, didecyldimethylammonium chloride

## Abstract

The foodborne pathogen, *Listeria monocytogenes*, (*Lm*), frequently undergoes selection pressure associated with the extensive use of disinfectants, such as quaternary ammonium compounds, which are widely used in food processing plants. The repeated exposure to sub-inhibitory biocide concentrations can induce increased tolerance to these compounds, but can also trigger the development of antibiotic resistance, and both increase the risk of food contamination and persistence in food production environments. Although the acquisition of genes can explain biocide tolerance, the genetic mechanisms underlying the adaptive cross-resistance to antibiotics remain unclear. We previously showed that repeated exposure to benzalkonium chloride (BC) and didecyldimethyl ammonium chloride (DDAC) led to reduced susceptibility to ciprofloxacin in *Lm* strains from diverse sources. Here, we compared the genomes of 16 biocide-adapted and 10 parental strains to identify the molecular mechanisms of fluoroquinolone cross-resistance. A core genome SNP analysis identified various mutations in the transcriptional regulator *fepR* (lmo2088) for 94% of the adapted strains and mutations in other effectors at a lower frequency. FepR is a local repressor of the MATE fluoroquinolone efflux pump FepA. The impact of the mutations on the structure and function of the protein was assessed by performing *in silico* prediction and protein homology modeling. Our results show that 75% of the missense mutations observed in *fepR* are located in the HTH domain of the protein, within the DNA interaction site. These mutations are predicted to reduce the activity of the regulator, leading to the overexpression of the efflux pump responsible for the ciprofloxacin-enhanced resistance.

## Introduction

*Listeria monocytogenes* (*Lm*) is a Gram-positive intracellular bacterium responsible for a serious food-borne zoonosis called listeriosis. This pathogen is transmissible to humans through the consumption of contaminated food. In 2019, listeriosis was the most serious zoonosis with the highest case-fatality rate (8.9%) among outbreak-related illnesses and an increasing incidence in this disease has been reported throughout European countries (EFSA and ECDC, 2021). Treatment with antibiotics is generally required for the treatment of listeriosis, and faced with the global increase in antibiotic resistance in bacteria, including Lm, there is a need to better understand the factors responsible for antibiotic resistance selection (Olaimat et al., 2018).

Contamination of food during processing is recognized as the main transmission route of Lm. The ability of Lm to adapt to stress, grow at low temperatures, form biofilms and then persist in food processing plants for years has made this bacterium a major challenge for food safety (Wiktorczyk-Kapischke et al., 2021). Successful control of *Lm* in the food chain requires appropriate cleaning and sanitation programs. Biocides play an essential role in limiting the dissemination of bacterial pathogens, and quaternary ammonium compounds (QACs), such as benzalkonium chloride (BC), are widely used as disinfectants in various food sectors (Gerba, 2015). To ensure effective killing of the foodborne pathogens, these biocides are often used at a concentration higher than the minimum bactericidal concentration. However, traces of these molecules may be found in different processing environments over time and the effect of sub-lethal concentration of biocide on bacterial cells is not very well understood.

The development of bacterial resistance to QACs (Lunden et al., 2003; Yu et al., 2018) and the subsequent effects on antibiotic susceptibility have been reported in several studies (Rakic-Martinez et al., 2011; Yu et al., 2018; Kode et al., 2021; Bland et al., 2022). We recently demonstrated a link between QAC exposure and reduced susceptibility to a fluoroquinolone antibiotic in *Lm*, using a panel of 28 strains from different sources and different clonal complexes (CC) (Guérin et al., 2021). Repeated exposure to sub-lethal concentrations of BC or to another QAC compound, didecyldimethylammonium chloride (DDAC), recurrently led to the significant decrease of susceptibility to ciprofloxacin (CIP) in 14 and 21 *Lm* strains, respectively. Nevertheless, the molecular mechanisms behind QAC adaptation and CIP resistance remain unclear and dedicated studies are needed to better understand genetic pathways involved in this cross-resistance. That indeed constitutes a prerequisite (i) to determine the mechanisms of antibiotic cross-resistance in biocide-adapted strains, (ii) to better assess the risk associated with the use of biocides in relation to antibiotic resistance and ultimately, and (iii) to develop marker-based surveillance tools.

Therefore, relying on QAC-adapted CIP resistant *Lm* strains previously obtained (Guérin et al., 2021), we sequenced and compared the whole genome of 16 biocide-adapted strains, 10 parental strains and 10 control strains to determine the genetic mechanisms underlying the adaptation of the *Lm* strains to BC and DDAC and the associated reduction in susceptibility to CIP.

## Materials and Methods

### Panel of Strains

The 36 strains used in this study were obtained in a previous study (Guérin et al., 2021) and are described in Table 1. Briefly, 10 initial, “parental” strains had been isolated from three different sources (food products, animal samples, and the natural environment) from five different countries and belonging to eight distinct CCs (two from lineage I and six from lineage II) (Table 1). These 10 strains were exposed to sublethal concentrations of two QACs (BC at 0.6 mg.L^–1^ and DDAC at 0.31 mg.L^–1^) for 10 consecutive days, as described in detail by Guérin et al. (2021). The minimal inhibitory concentrations (MIC) of ciprofloxacin was determined after the adaptation by the broth microdilution method using concentration ranging from CIP, 0.25 to 32 mg.L^–1^. Sixteen “adapted” strains (seven BC and nine DDAC) showed a significant increase in CIP resistance (Table 1). Finally, the 10 “control” strains were the 10 parental strains exposed to sterile water instead of the biocide solution.

**TABLE 1:** Description of the 10 *Listeria monocytogenes* parental strains and the corresponding strains adapted to the two biocides, benzalkonium chloride (BC), and didecyldimethylammonium chloride (DDAC), or exposed to water (referred to as Control).

Strain	Listadapt id^a^	Source category	Lineage	CC	CIP MIC^c^ (mg/L)	Biocide adaptation	
						DDAC	BC
Parental	FR-DA-B-CH-295	F	II	CC21	1		
Control	FR-BIO-T-500				1		
Adapted	FR-BIO-DC-501				8	X	
Parental	FR-ME-U-UN-465	F	I	CC2	2		
Control	FR-BIO-T-502				2		
Adapted	FR-BIO-BC-503				8		X
Adapted	FR-BIO-DC-504				16	X	
Parental	FR-ME-P-UN-410	F	II	CC37	2		
Control	FR-BIO-T-507				1		
Adapted	FR-BIO-DC-508				8	X	
Parental	FR-VE-U-UN-470	F	II	CC37	2		
Control	FR-BIO-T-505				2		
Adapted	FR-BIO-DC-506				8	X	
Parental	CZ-NAT-SO-22	NE	I	CC1	2		
Control	CZ-BIO-T-276				2		
Adapted	CZ-BIO-BC-277				8		X
Adapted	CZ-BIO-CD-278				8	X	
Parental	NO-DEE-FE-3	A	II	CC11	1		
Control	NO-BIO-T-40				2		
Adapted	NO-BIO-BC-41				4		X
Parental	CZ-OTH-UN-8	A	II	CC7	2		
Control	CZ-BIO-T-282				2		
Adapted	CZ-BIO-BC-283				8		X
Adapted	CZ-BIO-CD-284				16	X	
Parental	CZ-NAT-SO-15	NE	II	CC20	2		
Control	CZ-BIO-T-286				2		
Adapted	CZ-BIO-BC-287				8		X
Adapted	CZ-BIO-CD-288				8	X	
Parental	IT-FOX-FE-63	A	I	CC1	4		
Control	IT-BIO-T-137				2		
Adapted	IT-BIO-BC-138				8		X
Adapted	IT-BIO-CD-139				8	X	
Parental	NL-GOA-UN-2	A	II	CC26	2		
Control	NL-BIO-T-65				2		
Adapted	NL-BIO-BC-66				8		X
Adapted	NL-BIO-CD-67				8	X	

*^a^The strains were isolated as part of the European project “LISTADAPT” (Adaptive traits of Listeria monocytogenes to its diverse ecological niches; https://onehealthejp.eu/jrp-listadapt/).*

*CZ, Czech Republic; NO, Norway; NL, the Netherlands; FR, France; IT, Italy.*

*^b^F, Food; A, Animal; NE, Natural environment.*

*^c^Ciprofloxacin minimum inhibitory concentration (CIP MIC).*

### Genome Sequencing

Genomic DNA of the 36 *L. monocytogenes* strains was extracted using the Wizard^®^ Genomic DNA Purification Kit (Promega, France) according to manufacturer’s instructions for Gram-positive bacteria. Genome libraries were sequenced by paired-end reads (2 × 150 bp) at the ‘‘Institut du Cerveau et de la Moëlle’’ (ICM, France)^1^ using the Illumina NovaSeq 6000 sequencing system. The raw reads were processed using the harmonized in-house workflow ARTWork.^2^ This pipeline performs various analyses (variant calling, *de novo* assembly, annotation, quality control, read contamination) that have been described in detail in previous studies (Vila Nova et al., 2019; Palma et al., 2020). Sequence type (ST) and CC were also predicted based on the Listeria Multilocus ST (MLST) scheme (Moura et al., 2016). The accession numbers, the typing data, the sequencing coverage and the metrics of the genome assemblies are provided in Supplementary Table 1. All the reads passed the quality check steps defined in ARTwork and inter-and intra-species contamination was not detected.

### Genomic Analyses

Single nucleotide polymorphisms (SNPs) were predicted by aligning the reads of the adapted strains and control strains against a reference parental genome assembly using Snippy (V. 4.6.0)^3^ with default parameters. The type of variant (SNP/insertion/deletion) and the effect of the mutation (missense_variant, stop_lost&splice_region_variant and stop_gained) was provided in the Snippy report. The SNPs shared between the adapted and corresponding control strains were filtered because these mutations were not associated with the cross-resistance phenotype.

The annotated genome feature format (GFF) files produced by Prokka v1.13.3 (Seemann, 2014) were used to identify the pangenome of the 36 genomes through the Panaroo workflow (Tonkin-Hill et al., 2020) in strict mode for maximal contamination removal. Differences observed in the gene content between parental and control or adapted strains were characterized with a BLAST analysis.

### Prediction of *fepR* Protein Structure

FepR is a transcriptional regulator of the fluoroquinolone efflux pump (FepA). Based on the different FepR amino acid sequences, the protein structure of the regulator FepR was predicted by homology using the SWISS-MODEL application (Waterhouse et al., 2018)^4^ based on the highest scoring template (Lmo2088 from EGD-e accession: 5ZTC on https://www.rcsb.org/structure/5ZTC) (Samad et al., 2019). Model quality was estimated as recommended in Studer et al. (2019).

Domains were predicted using the InterProScan (Jones et al., 2014)^5^ and PROSITE (De Castro et al., 2006)^6^ webtools. Ligand-binding pockets were predicted using P2rank software (Krivák and Hoksza, 2018). Ligand docking was evaluated using the COACH-D online tool (Wu et al., 2018). To further explore the docking site, the secondary structure of the fepA promoter region (76 bp) extracted from EGD-e sequence (NC_003210.1) was generated using the DNA Sequence to Structure tool (available at http://www.scfbio-iitd.res.in/software/drugdesign/bdna.jsp) and docked on the helix-turn-helix (HTH) domain of FepR using the HADDOCK web server (V. 2.4) (Van Zundert et al., 2016). The protein surface was visualized using pymol.^7^

## Results

### Single Nucleotide Polymorphism Identification

The comparison of the whole genome sequences between the 16 adapted strains and their parental strains identified a total of 37 SNPs and 3 deletion sequences in 18 genes as well as 10 SNPs and two insertion sequences in 11 intergenic regions (Supplementary Table 2). Overall, 18 and 21 mutations were observed in the strains adapted to BC and DDAC, respectively, and the number of mutations in each strain varied from one (CZ-BIO-DC-288) to seven mutations (FR-BIO-DC-501).

Among the 18 mutated genes, 13 encoded proteins of known functions and five encoded hypothetical proteins (Table 2). Our results revealed that mutations in the *fepR* gene were detected in 15 out of the 16 adapted strains, in contrast to the other genes that were identified at much lower frequencies. The *fepR* gene encodes a *tetR*-like transcriptional regulator that represses the activity of the multidrug effux pump FepA. The 15 mutations were spread out in 14 different positions in *fepR*. Six of these were non-sense mutations resulting in the insertion of a premature stop codon (PMSC) (six strains), one was a frameshift mutation (one strain), while missense mutations were identified for the eight remaining strains (Table 3).

**TABLE 2:** Genes presenting mutations in the 16 adapted strains [7 strains adapted to benzalkonium chloride (BC) and 9 strains adapted to didecyldimethylammonium chloride (DDAC)].

Gene	Product	EGD-e locus	Frequency^a^	BC	DDAC
-	Hypothetical protein	Absent in EGD-e	2	1	1
*inlJ*	Internalin-J precursor	Absent in EGD-e	1	-	1
-	Hypothetical protein	Absent in EGD-e	1	-	1
-	Hypothetical protein	Absent in EGD-e	1	-	1
-	Hypothetical protein	Absent in EGD-e	2	1	1
-	Hypothetical protein	Absent in EGD-e	1	1	-
*nagC*	N-acetylglucosamine repressor	lmo0178	1	-	1
*ftsH*	ATP-dependent zinc metalloprotease FtsH	lmo0220	1	-	1
*walR*	Transcriptional regulatory protein WalR	lmo0287	3	2	1
*walK*	Sensor histidine kinase WalK	lmo0288	1	1	-
*manZ*	PTS system mannose-specific EIID component	lmo0781	1	1	-
*rsbS*	RsbT antagonist protein RsbS	lmo0890	2	2	-
*rsbU*	Phosphoserine phosphatase RsbU	lmo0892	3	2	1
*mntH*	Divalent metal cation transporter MntH	lmo1424	1	-	1
*murG*	UDP-N-acetylglucosamine–N-acetylmuramyl- (pentapeptide) pyrophosphoryl-undecaprenol N-acetylglucosamine transferase	lmo2035	1	1	-
*fepR*	HTH-type transcriptional regulator AcrR	lmo2088	15	6	9
-	Internalin	lmo2396	1	-	1
*kdpD*	Sensor protein KdpD	lmo2679	1	-	1

*^a^The frequency is defined here as the number of strains showing a mutation in this gene.*

**TABLE 3:** Amino acid changes in the FepR protein.

Listadapt id	Position/amino acid change in FepR														Biocide adaptation
EGD-e (lmo2088)	19	22	27	37	44	46	100	103	108	116	121	130	157	168	
	Phe	Thr	Ile	Ser	Asp	Asp	Trp	Tyr	Pro	Gln	Glu	Phe	Gly	Tyr	
FR-BIO-DC-501		Ile													DDAC
FR-BIO-BC-503													Stop		BC
FR-BIO-DC-504							FS								DDAC
FR-BIO-DC-508								Stop							DDAC
FR-BIO-DC-506											Stop				DDAC
CZ-BIO-BC-277					Tyr										BC
CZ-BIO-CD-278										Stop					DDAC
NO-BIO-BC-41												Leu			BC
CZ-BIO-BC-283			Thr												BC
CZ-BIO-CD-284	Leu														DDAC
CZ-BIO-BC-287						Gly									BC
CZ-BIO-CD-288							Stop								DDAC
IT-BIO-CD-139				Tyr											DDAC
NL-BIO-BC-66									Leu						BC
NL-BIO-CD-67														Stop	DDAC

*Positions in orange correspond to the HTH domain and position in green locates in the vicinity of the ligand-binding pocket. Missense mutations are indicated in blue and the frameshift (FS) and premature stop codon (Stop) are indicated in red.*

The BC-adapted strain IT-BIO-BC-138 that did not display mutation in the *fepR* gene contained a single missense mutation (319A319T/Asn107Tyr) in the transcriptional regulator *walR*. This gene encodes a two-component response regulator involved in membrane metabolism and antibiotic resistance. Mutations in this gene were identified in two other strains (NL-BIO-BC-66 and NL-BIO-DC-67) (Table 2).

The other genes in which mutations were observed were mainly stress/antibiotic-resistance-related genes (*rbsS*/*rsbU* and *kdpD)* and genes involved in cell wall metabolism (*nagC*, *manZ*, *murG*). Some of these genes were only detected in BC- (*walK*, *manZ*, *rsbS*, and *murG*) or in DDAC-adapted (*nagC* and *kdpD*) strains while five mutated genes (*walR*, *rbsU*, *fepR*, and two genes encoding hypothetical proteins) were identified in both (Table 2 and Supplementary Table 2).

### Investigation of *fepR* Mutations

The impact of the eight missense mutations on the structure and function of the protein was analyzed by performing *in silico* prediction and protein homology modeling.

**FIGURE 1:**
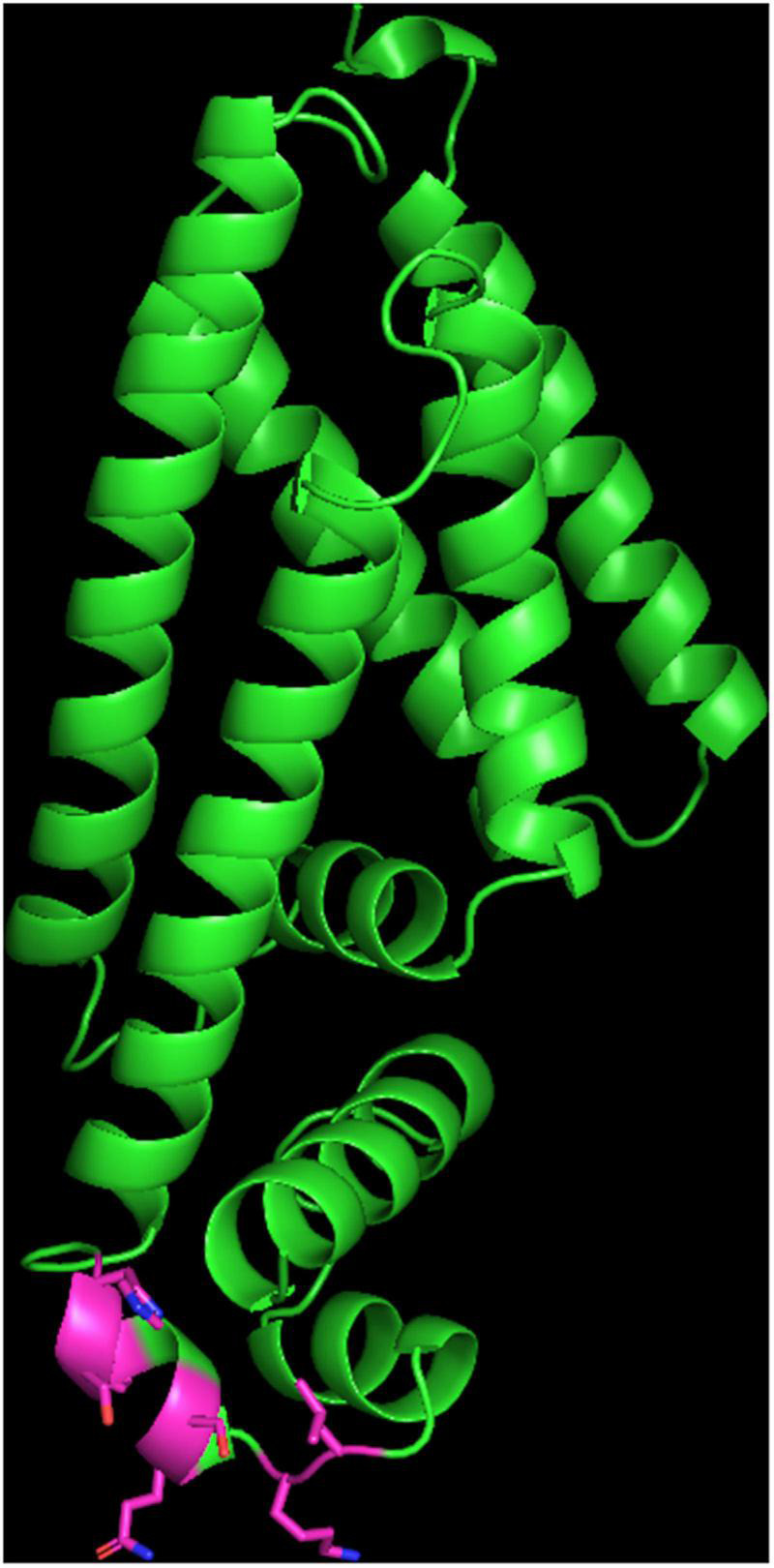
Structure of the FepR protein. The carbon of the side chain of the mutated residues inside the HTH domain are shown in pink. Oxygen atoms are shown in red and nitrogen in blue. Hydrogen atoms are hidden.

The quality parameters are reported in Supplementary Table 3. Most mutations (6 out of 8) were located in the HTH domain of the protein located in residues 19, 22, 27, 37, 44, and 46 and are predicted to significantly modified the protein surface in this area (Figure 1). These changes potentially reduce the capacity of FepR to bind DNA leading to an upregulation of the *fepA* gene.

**FIGURE 2:**
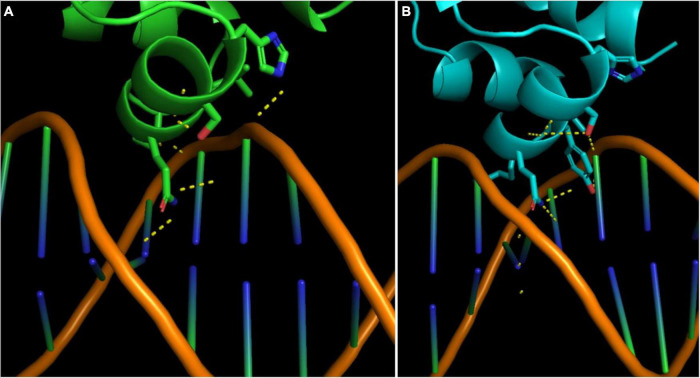
Impact of the Ser37Tyr mutation observed in the DDAC-adapted IT-BIO-DC-139 strain on DNA binding. Carbon atoms are shown in green for the parental strain (IT-FOX-FE-63) **(A)** and in blue for the DDAC-adapted strain IT-BIO-DC-139 **(B)**. The calculated hydrogen bonds are indicated by a yellow dotted line. Only the scaffold is shown for the double-stranded DNA (dsDNA) molecule. The dsDNA is docked in the parental strain; the modification of the residue 37 alters the conformation of the protein at the DNA-binding site, leading to a reduction of affinity for the DNA strands (affinity score of –82.9 compared with –104.2 from the *fepR* model of the parental strain). (DDAC, didecyldimethylammonium chloride).

**FIGURE 3:**
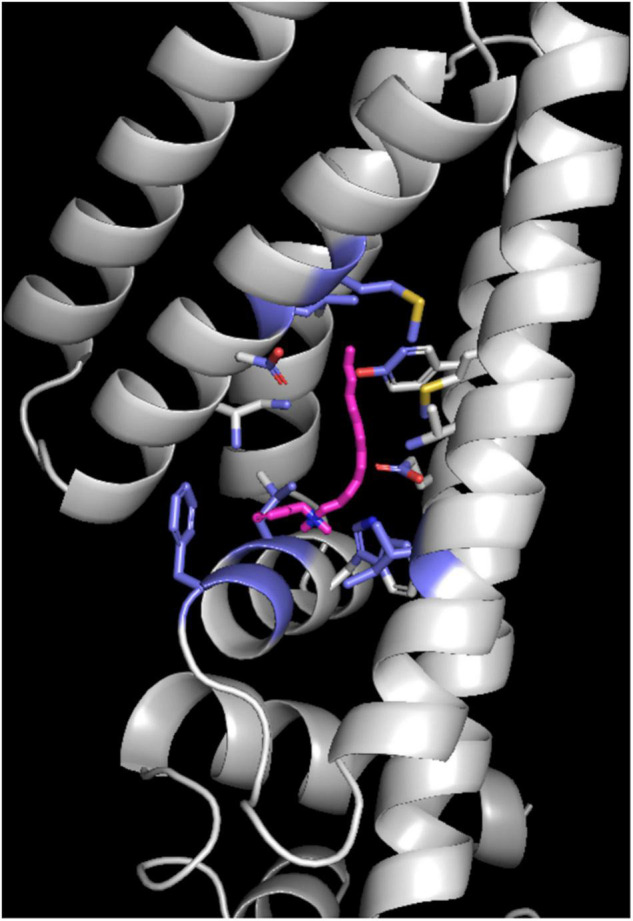
Docking of benzalkonium chloride (pink) in the predicted pocket of FepR using the P2Rsankweb application. The predicted interacting residues are shown in violet. Oxygen atoms are shown in red, nitrogen in blue, sulfur in yellow.

Based on the main allele of *fepR*, DNA interaction was predicted to occur at two locations inside the HTH domain: between residues 1, 23, 24, 35, 39, 44, and 45 and between residues 2, 33–37, 40, and 41 (confidence score 1) (Figure 2). Docking was attempted with a dsDNA sequence extracted from the promoter region of EGD-e. The best binding pose predicted that the interacting residues were 33, 34, 36, 37, 39, 40, and 41 (Figure 2A). The mutations detected in residues 19, 22, 27, 37, 44, and 46 may have a significant impact on protein binding to DNA at the recognition sequence reducing the efficiency of this regulator (Figure 2B). The docking analysis showed a reduction of the HTH domain affinity for the *fepA* promoter sequence for these six mutations (affinity score from –95.2 to –82.9) in comparison to the affinity score of –104.2 for the native fepR structure (Supplementary Table 3). To investigate the affinity of FepR to QACs, pocket prediction was performed and a pocket was predicted in the vicinity of neighboring residues 60, 100, 101, 104, 105, 119, 123, 126, 156, 159, 160, and 163 with an affinity of –6.3 for BC and DDAC (Figure 3). The two remaining mutations did not seem to affect the binding of these ligands either because the mutation is located far from the pocket (Pro108Leu) or because it did not seem to affect the affinity for BC or DDAC (Phe130Leu).

### Pangenome

The pangenome of the control and adapted strains was compared with that of the parental strains to detect potential variations in gene content. The different presence-absence matrices and summary statistics generated by Panaroo are available in Supplementary Table 4.

A deletion of 62 genes was observed in the BC-adapted strain FR-BIO-DC-506. This 41 kb region shares 93.5% identity (72% coverage) with the *L. monocytogenes* prophage LP-101 (NC_024387.1) (Denes et al., 2014). The prophage identified in this study is integrated in the parental and control genomes at the tRNA-Arg locus. The analysis also revealed that 83 genes were missing in the adapted strain CZ-BIO-BC-287. The element carrying these genes displayed a strong homology (>99% identity and coverage) with the pLmN1546 plasmid isolated from the *Listeria monocytogenes* strain Lm N1546 (CP013725.1) (Tasara et al., 2016). This 86 kb plasmid encoding the cadmium-resistance transcriptional regulatory protein CadC was present in all the other strains derived from the parental strain. A similar plasmid was also lost by the control and BC-adapted strains NL-BIO-T-65 and NL-BIO-BC-66, respectively. The loss of this plasmid in the control strain indicates it is not linked with the resistance phenotype. A few additional missing genes encoding hypothetical protein, transposase and internalin are indicated in Supplementary Table 4.

## Discussion

CIP belongs to the fluoroquinolone family, a family of antibiotics extensively used in human medicine. Different types of selective pressure (such as antibiotics, biocides, or heavy metals) may play a role in the prevalence of antibiotic resistance of *Lm* in the food chain. Exposure to disinfectant biocides such as the QACs BC and DDAC, extensively used in the food processing industry, has the potential to promote cross-resistance to CIP, as indicated by an increasing number of studies (Rakic-Martinez et al., 2011; Yu et al., 2018; Guérin et al., 2021; Kode et al., 2021; Bland et al., 2022). These biocides may co-select for strains resistant to CIP, due to shared mechanisms of resistance (cross-resistance) (Kampf, 2019). However, very limited information is currently available on the genetic mechanisms underlying CIP resistance following exposure to QACs. Here, we studied this issue by analyzing genetic traits linked to this cross-resistance.

The most striking finding of this study was the recurrence of the mutations observed in *fepR*, the multi-antimicrobial extrusion (MATE) transporter (*fepA*) regulator for all the CIP resistant DDAC-adapted strains and for six out of the seven CIP resistant BC-adapted strains. This is consistent with very recent findings showing that mutations in *fepR* play a role in the reduction in antibiotic susceptibility including CIP following low-level adaptation to a commercial sanitizer containing QAC (Bland et al., 2022). Interestingly, overexpression of the MATE efflux pump FepA through inactivation of the fepR repressor has been described to be responsible for fluoroquinolone resistance in *Lm* (Guérin et al., 2014). The involvement of efflux pump systems in adaptation to QAC and CIP resistance found here is also consistent with previous studies reporting their involvement in cross-resistance between various biocides (especially QAC) and antibiotics in various pathogenic Gram-positive and Gram-negative species including *Lm*, *Staphylococcus aureus*, *Escherichia coli*, and *Salmonella enterica* (Buffet-Bataillon et al., 2016). Nevertheless, this study is the first to report the *fepR* gene as a central target in the adaptation to DDAC and cross-resistance development to CIP in *Lm*.

The FepR protein contains an HTH domain in aa residues 1–60 (predicted through InterProScan and PROSITE) and a tetracyclin repressor-like C-terminal domain in residues 99–192. The analysis of the protein structure revealed that 75% of the missense mutations were located in the DNA interaction site within the HTH domain thus reducing the affinity of FepR for the *fepA* promoter region and leading to the overexpression of the efflux pump FepA responsible for the reduced susceptibility to CIP.

The mutation Glu21PMSC observed by Guérin et al. (2014) in a clinical *Lm* isolate was also detected inside the DNA interaction site. qRT-PCR analysis demonstrated the overexpression (64-fold higher) of the MATE efflux pump *fepA* in the mutant strain and a clean *fepR* deletion mutant, confirming the role of FepR as a local repressor of FepA (Guérin et al., 2014). Another mutation has been reported by Wilson et al. (2018) in one CIP-resistant CC1 strain, isolated from food production supplies in Australia (Wilson et al., 2018). This mutation was located at nucleotide position 181 and resulted in a PMSC in place of aspartic acid (Asp61PSMC). More recently, Bland et al. (2022) identified five mutations (His41Tyr/Leu96PMSC/Pro107Gln/Pro108Ser/Glu176PMSC) in five strains belonging to three hypervirulent CCs (Bland et al., 2022). All these mutations are different from those found in this study. Our results also demonstrate that *fepR* mutations occurred independently of the genetic background (CC) or the source, as the 15 strains developing cross-resistance were very diverse.

In addition to the recurrent mutations observed in *fepR*, other genes were mutated in QAC-adapted CIP-resistant strains. Mutations in the *walR* and *walK* genes were identified in four QAC-adapted CIP resistant strains. The two-component system is known to regulate cell wall metabolism, virulence regulation, biofilm production, oxidative stress resistance, and antibiotic resistance via direct or indirect regulation of autolysins (Takada and Yoshikawa, 2018). Interestingly, the BC-adapted strain IT-BIO-BC-138 was the only strain that did not display mutation in *fepR* but a missense mutation in *walR*. This result suggests that such mutation is responsible for the CIP resistance observed in IT-BIO-BC-138 and that WalR regulator could be another target for QAC adaptive CIP cross-resistance selection.

Some QAC-adapted CIP-resistant strains also displayed mutations in the *rsbS* or *rsbU* genes, both involved in the signal transduction responsible for the regulation of alternative sigma factor σ^B^, which is known as a mediator of the general stress response in *Lm* (Guerreiro et al., 2020). These mutations may thereby participate in the CIP resistance observed in QAC-adapted strains through the upregulation of stress response factors acting on various cellular processes including cell envelope modification, pH homeostasis, osmoregulation, quorum sensing, or antibiotic resistance (Guerreiro et al., 2020).

## Conclusion

In conclusion, several genetic markers associated with CIP cross-resistance in QAC-resistant Listeria have been identified. In particular, the DNA binding region of the FepR regulator appears to be an important target in CIP cross-resistance. In the present study, the mutations observed in the FepR HTH domain are predicting to affect the promoter activity of *fepA* and resulting in the deregulation of the efflux pump FepA, responsible for the cross-resistance phenotype. Other stress-related genes such as the transcriptional regulator WalR may contribute to the CIP resistance mechanism and will need further attention.

## Data Availability Statement

The datasets presented in this study can be found in online repositories. The names of the repository/repositories and accession number(s) can be found in the article/Supplementary Material.

## Author Contributions

P-ED contributed substantially to data analysis, quality control of the genomic dataset, writing, and editing the manuscript. YS contributed to study design, acquisition of strains and the corresponding genomes, data analysis as well as writing, and editing the manuscript. PL provided technical assistance. CS participated in study design and contributed to writing and editing of the manuscript. AB provided technical assistance and participated in designing the study and in writing and revising the manuscript. SR designed, coordinated the study, and contributed to writing and editing the manuscript. All authors read and approved the final manuscript.

## Conflict of Interest

The authors declare that the research was conducted in the absence of any commercial or financial relationships that could be construed as a potential conflict of interest.

## Publisher’s Note

All claims expressed in this article are solely those of the authors and do not necessarily represent those of their affiliated organizations, or those of the publisher, the editors and the reviewers. Any product that may be evaluated in this article, or claim that may be made by its manufacturer, is not guaranteed or endorsed by the publisher.

## Footnotes

1
https://icm-institute.org/fr/

2
https://github.com/afelten-Anses/ARtWORK

3
https://github.com/tseemann/snippy

4
https://swissmodel.expasy.org/interactive

5
https://www.ebi.ac.uk/interpro/search/sequence/

6
https://prosite.expasy.org/

7
https://pymol.org/2/
